# Antibacterial activity of endemic Satureja Khuzistanica Jamzad essential oil against oral pathogens

**Published:** 2009-01-07

**Authors:** Sogol Seghatoleslami, Nasrin Samadi, Ali Salehnia, Shahram Azimi

**Affiliations:** 1 *General Dentist, Tehran, Iran.*; 2 *Department of Drug and Food Control Department, Faculty of Pharmacy, Biotechnology Research Center, Tehran University of Medical Sciences, Tehran, Iran.*; 3 *Pharmacist, Khorraman Company, Khorramabad, Lorestan, Iran.*; 4 *Department of Endodontics, Dental School, Islamic Azad University of Medical Sciences, Tehran, Iran.*

**Keywords:** Antibacterial activity, Carvacrol, Essential oil, *Satureja Khuzistanica* Jamzad.

## Abstract

**INTRODUCTION:** To assess the antibacterial effects of an Iranian endemic essential oil, *Satureja Khuzistanica* Jamzad (SKJ) when used as an intracanal antiseptic and interappointment medicament.

**MATERIALS AND METHODS:** Antimicrobial activity and minimum inhibition concentrations (MICs) of SKJ essential oil with and without calcium hydroxide (CH) against eleven aerobic, microaerophilic and anaerobic bacteria were assessed. The evaluation was carried out by agar dilution and well diffusion methods. The results were measured and recorded by an independent observer. Data were analyzed statistically using student t-test.

**RESULTS:** The MIC for eight species was recorded in 0.31 mg/mL of essential oil. *Pseudomonas aeruginosa* with a MIC value of 1.25 mg/mL appeared to be the most resistant bacterium; while only 0.16 mg/mL of essential oil was sufficient to inhibit the growth of *Bacillus subtilis *and* Staphylococcus aureus.* The inhibition zone of the antiseptic oil (at 0.31 mg/mL) with *E. faecalis *in the well diffusion method was 13 mm; this was comparable with 12.5 mm inhibition zone value of the tetracycline disc (30 µg). No synergistic effect was found in combination of essential oil and CH powder.

**CONCLUSION:** SKJ essential oil with the concentration of 0.31 mg/mL is effective against most of oral pathogens including *E. faecalis*.

## INTRODUCTION

Persistent infection after endodontic therapy has been considered the main etiologic factor in treatment failure (1). The number of bacterial species in root canals may vary from 1 to 12, and the number of bacterial cells recovered between ˂10^2 ^to ˃10^8^ (2). The root canal environment after chemomechanical treatment becomes unfavorable for microorganisms; there is reduced oxygen tension, limited nutrient availability and antimicrobial agents that act as driving forces in survival balance of bacteria in the root canal system (3). Teeth with primary infection have higher numbers of black pigmented Gram-negative anaerobes; while root filled teeth with periradicular lesions have significantly fewer black pigmented rods and have more Gram-positive bacteria (4). Root canal dentinal tubules harbor microorganisms; also bacterial biofilm may be present at the apical portion of root canal and extra radicular regions (5). Therefore, irrigation with broad spectrum antiseptic substances and interappointment intracanal medication has become a standard regimen in root canal therapy.

Many species and herbs exert antimicrobial activity due to their essential oil fractions. For thousands of years clove oil, (eugenol) has been used in dentistry. Creosote, which contains several phenolic compounds such as guaiacol and cresol, is also used for the sedation of inflamed dental pulps (6,7). The antimicrobial activity of essential oils is due to a number of small terpenoids and phenol compounds (8), several of these are classified as Generally Recognized as Safe (GRAS) (9). The antimicrobial activities of oregano, savory and thyme were first reported during 1950s (10). Recent studies have shown oregano, thyme, clove and cinnamon to be among the most active antimicrobials (11). Gram-negative bacteria were shown to be generally more resistant than Gram-positive bacteria to the effects of essential oils because of the lipopolysaccharide of their outer membrane (10). *Satureja khuzistanica *Jamzad (SKJ; Lamiaceae family), is an endemic plant the southern part of Iran and has been traditionally used as an analgesic and antiseptic among nomadic inhabitants. Chemical analysis revealed that the major constitutes of SKJ were carvacrol (93%), eugenol (1.0%), P-cymene (0.8%) and thymol (0.6%) and other trace compounds (12). Carvacrol (2-methyl-5-(1-methylethyl)-phenol) is generally recognized as a safe food additive and is used as a flavoring agent in baked goods, sweets and beverages. Carvacrol has inhibitory and biocidal effects on range of bacteria including *Escherichia coli*, *Bacillus cereus*, *Listeria monocytogenes*, *Salmonella enterica*, *Clostridium jejuni* and *Lactobacillus sakei* (11).

The search for a natural antimicrobial substance with less side effects is warranted; this is because many of the currently used drugs have adverse side effects; some are harmful and some ineffective. The aim of this study was to determine the antimicrobial activity of SKJ essential oil against oral pathogens including aerobic, microaerophilic and anaerobic bacteria.

## MATERIALS AND METHODS

The minimum inhibition concentration (MIC) of SKJ essential oil (Khorraman Co., Lorestan, Iran) was determined by conventional agar dilution method with respect to different microorganisms including *Enterococcus faecalis* ATCC 29212, *Enterococcus faecalis* ATCC 8213, *Pseudomonas aeruginosa* ATCC 9027, *Escherichia coli* ATCC 8739, *Staphylococcus epidermidis* ATCC 12228, *Staphylococcus aureus* ATCC 6538, *Bacillus subtilis* ATCC 6633, *Streptococcus sanguis* ATCC10556, *Streptococcus mutans* ATCC 35668, *Actinomyces viscosus* PTCC 1202, *Propionibacterium freudenrechi* ATCC 6207. The microorganisms were retrieved from Department of Drug and Food Control, School of Pharmacy, Tehran University of Medical Sciences.

Two-fold dilution of the essential oil was prepared in dimethylsulfoxide (DMSO; 1 mL). Each dilution was then added to 19 mL of a suitable molten agar medium (Table 1) to obtain the final concentrations (20 to 0.018 mg/mL). The bacterial suspension was prepared by suspending refreshed colonies of each bacterium in 0.9% saline. The inocula were adjusted photometrically at 600 nm to a cell density equivalent to approximately 0.5 McFarland standards (1.5×10^8 ^CFU/mL). The suspensions were then diluted in 0.9% saline to obtain 10^7^ CFU/mL. The plates were spot-inoculated with 1 µL of each prepared bacterial suspension (10^4 ^CFU/spot); including a control plate containing 1 mL DMSO without essential oil. The aerobic microorganisms were incubated at 30-35^o^C and the plates containing *S. sanguis* and *S*. *mutans* were incubated at 30-35˚C and 5% CO_2_ for 18-24 hours. The *P. bacterium* was incubated in anaerobic conditions (anaerobic Jar, MART system) at 30-35˚C for 48 h. The lowest concentration of the antimicrobial agent that completely inhibits visible growth of the bacteria was recorded as the MIC value.

***Well Diffusion Assay:*** Tryptic soy agar** (**TSA) plates were seeded with *E. faecalis* ATCC 29212 suspension (1.5×10^8 ^CFU/mL) using a sterile cotton swab. Essential oil was dissolved in DMSO and diluted in a two-fold manner to provide the concentrations of 1.25 mg/mL to 0.05 mg/mL. Wells were prepared by punching a stainless steel cylinder into the agar plates and removing the agar to form a well. Then 80 µL aliquots of each dilution were placed in three independent wells. DMSO (80 µL) was placed in wells as the negative control.

Tetracycline discs (30 µg) were used as the standard antibacterial agent.

**Table 1:** MIC of *Satureja Khuzistanica *Jamzad essential oil against aerobic, microaerophilic and anaerobic microorganisms

**Microorganism**	**Culture medium**	**MIC mg/mL**
*Enterococcus faecalis* ATCC 29212	TSA^a^ and MHA^b^	0.31
*Enterococcus faecalis* ATCC 8213	TSA and MHA	0.31
*Pseudomonas aeroginosa* ATCC 9027	TSA and MHA	1.25
*Escherichia coli* ATCC 8739	TSA and MHA	0.31
*Staphylococcus epidermidis* ATCC 12228	TSA and MHA	0.31
*Staphylococcus aureus* ATCC 6538	TSA and MHA	0.16
*Bacillus subtilis* ATCC 6633	TSA and MHA	0.16
*Streptococcus sanguis* ATCC10556	Blood and BHI^c^ agar	0.31
*Streptococcus mutans* ATCC 35668	Blood and BHI agar	0.31
*Actinomyces viscosus* PTCC 1202	Blood and BHI agar	0.31
*Propionibacterium freudenrechi* ATCC 6207	Brucella agar	0.31

^a^ *: Tryptic soy agar;* ^b^ *: MHA, Muller-Hinton agar;* ^c^ *: BHI agar, Brain heart infusion agar*

**Table 2:** Antibacterial effect of SKJ essential oil (EO) dilutions and calcium hydroxide (CH) paste against *E*. *faecalis *by well diffusion assay

**compound**	**Mean IZD** ^a^ ** (mm)**	
	**EO **	**EO and CH**
Pure EO	-	29
EO 1.25 (mg/mL)	26	22
EO 0.62 (mg/mL)	18.5	19.5
EO 0.31 (mg/mL)	12.5	13
EO 0.16 (mg/mL)	0	0
EO 0.08 (mg/mL)	0	0
Tetracycline disc (30µg)	-	12.5
DMSO	-	0
CH paste	0	-

^a^ *: Inhibition zone diameter*

To evaluate the probable synergistic effect of essential oil in combination with pure calcium hydroxide powder, inhibition zones surrounding 5 dilutions of essential oil and calcium hydroxide were also measured. The antimicrobial activity of calcium hydroxide paste (made distilled water) was determined by placing 80 µL of paste in three wells individually. The plates were incubated at 30-35˚C for 18-24 h. After incubation the mean inhibition zone diameter for each concentration was determined.

***Statistical analysis:*** Means were compared with student's t-test at P<0.05.

## RESULTS

As shown in Table 1 the SKJ essential oil exhibited significant antimicrobial activity against all the bacteria. *Staphylococcus aureus* and* Bacillus subtilis* growth were inhibited at 0.16 mg/mL.* Pseudomonas aeruginosa* demonstrated the greatest resistance to SKJ with an MIC value of 1.25 mg/mL appeared to be the most resistant bacterium. Other aerobic, microaerophilic and anaerobic bacteria were inhibited at 0.31 mg/mL of essential oil.

The MIC value of SKJ was comparable to tetracycline discs; ie 0.31 mg/ml concentration of the oil resulted in 13 mm of bacterial inhibition, tetracycline demonstrated 12.5mm of inhibition (Table 2). The mean inhibition zone around pure SKJ essential oil was 30 mm while no inhibition zone was found around pure calcium hydroxide paste wells. No significant differences were found between inhibition zones obtained by essential oil alone or in combination with calcium hydroxide powder (P>0.05).

## DISCUSSION

A wide variety of medicaments have been used as intracanal antiseptics. Saturated calcium hydroxide (Ca(OH)_2_) paste, sodium hypochlorite (NaOCl), chlorhexidine (CHX), iodine potassium iodide (IKI), chlorine dioxide, phenolic compounds, formocresol, antibiotics such as tetracycline, and recently MTAD, are some examples (1) though there are certain concerns about their cytotoxicity and efficacy (13). Inhibitory effects of selected plant essential oils on the growth of pathogenic microorganisms is well documented and the phenolic components are chiefly responsible for such properties (10). An estimated 3000 essential oils are known, of which about 300 are commercially available (14). SKJ, an endemic plant of southern Iran, is known as stomachic, sedative and analgesic, especially for toothache among nomadic inhabitants. The chemical composition of the essential oil of SKJ, reveals carvacrol to be the major constituent in wild (93.9%) and cultivated (80.6%) plants (12). Such high concentration of carvacrol distinguishes *Satureja khuzistanica *Jamzad from other aromatic plants except for *Thymus* *capitatus *which contains 90% carvacrol and 10% thymol. Kandil *et al.* found essential oil of *Thymus* *Capitatus* to inhibit the growth of several bacteria and fungi (15). Amanlou *et al.* compared the antibacterial activity of crude methanolic extract of wild and cultivated SKJ and found the extract of wild plant to be stronger probably due to the presence of isoeugenol. The elimination of lipids and other components of this essential oil render it more effective than the methanolic extracts (16). The maximum activity of this plant extract was found to be against *Staphylococcus aureus *(2 mg/mL) and *Candida albicans* (1 mg/mL); Gram negative species, however, appear to be more resistant (16). Didry (1994) compared the antimicrobial activity of thymol, carvacrol, cinnamaldehyde and eugenol alone or combined on eight oral bacteria and found that the MIC values of the combined compounds were often lower. Eugenol displayed the most superior antimicrobial properties when combined with thymol or carvacrol (17). Methodological differences such as the solvent used, the concentration of essential oil, extraction technique, growth phase, culture medium, pH, temperature, incubation period are factors that make the comparison of published data challenging (14). Using different culture media such as Muller Hinton Agar (MHA) and Tryptic Soy Agar (TSA) for aerobic species, blood and BHI agar for microaerophilic bacteria and Brucella agar for anaerobic bacterium helped to obtain specific proliferative environments for each strain in our study. Interestingly, the MIC value for eight of tested species was 0.31 mg/mL. *Pseudomonas aeroginosa* was the most resistant strain among the listed bacteria with the MIC of 1.25 mg/mL.* E. faecalis* is the most common and occasionally the only single isolated bacterium from root canals with persistent periapical periodontitis (3). Therefore, *E. faecalis* ATCC 29212 was selected for additional well diffusion assay as the most resistant strain.

Despite some controversial reports regarding calcium hydroxide paste (1,13), it is still commonly used as an inter-appointment medicament. Interestingly, no inhibition zone was detected around pure calcium hydroxide plus distilled water wells after 24 h incubation period which confirms the ineffectiveness of the paste against *E. faecalis *when evaluated short term. Also there was no apparent synergy when calcium hydroxide and SKJ oil were mixed.

Essential oils are hydrophobic, a vital characteristic which enables them even in a low pH, to break through the lipids of the bacterial cell membrane and mitochondria, disturbing the structure and rendering them more permeable. Considering the large number of different groups of chemical compounds present in the essential oils, it is most likely that their antibacterial activity is not attributable to one specific mechanism rather against several targets in the bacterial cell (14). Stimulation of periapical repair following root canal treatment is a favorable property of any intracanal medication. Interestingly, with some phenolic compounds, an increase of the dehydrogenase activity was observed at concentrations lower than that which caused inhibition. Tsukamoto also reported that low concentrations of some phenolic compounds stimulated the proliferation of human pulpal fibroblasts. This phenomenon is known as “hormesis” which may contribute to periapical tissue repair (6,18).

No short and long-term *in vivo* toxicological data is available for carvacrol; whilst in vitro there is sufficient evidence to suggest that they exhibit mild to moderate toxic effects at the cellular level (14,19). Ipek *et al.* found carvacrol to possess a strong antimutagenic activity in human lymphocytes by inhibiting the induction of sister chromatid exchange formation and DNA synthesis in myoblast cells.

## CONCLUSIONS

The results of this study indicate that *Satureja khuzistanica *Jamzad essential oil can be used as an effective intracanal antiseptic solution at low and safe concentrations.
